# Characterization of the adaptive immune response in a mouse model for HPV-positive head and neck squamous cell carcinoma with implications to human disease

**DOI:** 10.1007/s00262-024-03907-y

**Published:** 2025-01-03

**Authors:** Franziska Oliveri, Linda Neher, Ronja Pscheid, Isabel Sewald, Sowmya Gowdavally, Annika C. Betzler, Jaqueline Hallitsch, Jens Greve, Simon Laban, Sebastian Schmid, Thomas K. Hoffmann, Patrick J. Schuler, Cornelia Brunner

**Affiliations:** 1 Department of Otorhinolaryngology, Head and Neck Surgery, Ulm University Medical Center, Ulm, Germany; 2 Department of Anesthesiology and Intensive Care Medicine, Ulm University Medical Center, Ulm, Germany; 3 Department of Otorhinolaryngology, University Hospital Heidelberg, Heidelberg, Germany; 4 Core Facility Immune Monitoring, Ulm University Medical Faculty, Ulm, Germany

**Keywords:** Head and neck cancer, HPV, Immune checkpoints, Tumor-infiltrating lymphocytes

## Abstract

Head and neck squamous cell carcinoma (HNSCC) is the seventh most common cancer worldwide with a poor prognosis for survival. Risk factors include alcohol and tobacco abuse and infection with human papilloma virus (HPV). To enhance anti-tumor immune responses immunotherapeutic approaches are approved for recurrent metastatic disease but only approx. 20% of patients respond to checkpoint blockade of the PD-1/PD-L1 axis. Therefore, preclinical research is needed to better understand molecular and cellular processes and to identify new therapeutic targets. Immunocompetent mouse models can serve these purposes but only few are currently available for HPV-positive HNSCC. Here, we established a mouse cell line overexpressing the oncogenes *E6*/*E7* of the HPV16 genome as well as a constitutively active form of H-Ras and studied the anti-tumor immune response upon orthotopic tumor growth at the floor of the mouth. Moreover, we analyzed the same immunoregulatory pathways in samples of HPV-positive cancer patients. T cells in the tumor of mice and humans exhibited high expression of CD39 and CD73, two ectoenzymes involved in the production of immunosuppressive adenosine from ATP, along with increased expression of PD-1, LAG-3 and GITR. Additionally, B cell responses were elevated in tumor-bearing mice, seen as an increase of germinal center, immunoregulatory marginal zone and follicular B cell subtypes. Taken together, this study suggests that the generated mouse model shares characteristics with human disease and can thus be used as a platform to study anti-tumor responses in HPV-positive HNSCC which will help to identify novel therapeutic targets.

**Supplementary Information:**

The online version contains supplementary material available at 10.1007/s00262-024-03907-y.

## Introduction

Head and neck squamous cell carcinomas (HNSCC) originate from the mucosal epithelium of the head and neck region including the oral cavity, larynx and pharynx. Besides alcohol and tobacco abuse, infection with carcinogenic human papilloma virus (HPV), particularly type 16 (HPV16), is a risk factor for cancer development with increasing incidence in recent years [1]. Due to the viral infection driving a strong immune response, HPV-positive HNSCC is associated with better prognosis for survival compared to HPV-negative HNSCC [2]. Main drivers of HPV-carcinogenesis are the viral oncogenes *E6* and *E7* which trigger cell cycle entry. Aberrant expression of *E6/E7* is associated with integration of viral genes into the human genome, thus enhancing carcinogenesis [1]. Additionally, HNSCC is characterized by genomic instability leading to accumulation of genomic mutations that contribute to cancer cell transformation, e.g. by affecting tumor suppressors [3, 4]. These factors contribute to the high incidence and poor 5-year overall survival rate of HNSCC, thus highlighting the need for new therapeutic options. Novel immune checkpoint inhibitors targeting the PD-1/PD-L1 axis have recently gained approval for recurrent metastatic disease, but only approx. 20% of patients responded to treatment [5, 6]. To identify and evaluate new therapeutic strategies, especially those involving the immune system, pre-clinical models are urgently needed but only few models exist for HPV-positive HNSCC [7, 8]. Therefore, this study aims at establishing a murine cell line overexpressing *HPV16-E6/E7* together with mutant H-Ras, similar to the model of Hoover et al*.* [7], and to characterize the induced adaptive immune response. Moreover, we analyzed human HPV-positive tumor samples to identify immunoregulatory pathways shared between mouse and human.

## Material and methods

### Generation of POECs to mimic HPV-positive HNSCC

Murine primary oral epithelial cells (POECs) were isolated from the oral cavity/pharyngeal region of C57BL/6 mice and cultured in keratinocyte growth medium 2 (Promocell) containing 2% fetal calf serum (FCS) and 1% penicillin/streptomycin. Retroviral vectors encoding for a H-Ras^V12S35^ (pBabe-puro-H-Ras^V12/S35^) and E6/E7 of the HPV16 genome (pLXSN16E6E7, addgene #52,394) were kindly provided by John Lee/Kimberly Lee [7] and used for transduction of POECs.

### Characterization of POEC-H-RAS.^V12S35^-E6/E7

Immunoblotting was performed as described in [9], using antibodies directed against H-Ras (Santa Cruz Biotechnology) and β-actin (Sigma-Aldrich).

RNA expression levels were analyzed using RNeasy Mini Kit, QuantiNova Reverse Transcription Kit and QuantiNova SYBR Green PCR Kit (all Qiagen) and primers to detect HPV16-E6 (F: 5’-CTGCAAGCAACAGTTACTGC-3’; R: 5’-GGCTTTTGACAGTTAATACACC-3’); HPV16-E7 (F: 5 ‘-CATGGAGATACACCTACATTG-3’; R: 5 ‘-CACACCCGAAGCGTAGAGTC-3 ‘); GAPDH (F: 5’-GAC TTCAACAGCAACTCCCAC-3’; R: 5’-TCCACCACCCTGTTGCTGTA-3’).

### Tumor induction

Male C57BL/6 mice were obtained from Janvier Biolabs and housed under specific pathogen-free conditions. 5 × 10^5^ POEC-H-RAS^V12S35^-E6/E7 cells were injected into the floor of the mouth under ketamine/xylazine anesthesia at 10–12 weeks of age. Mice were sacrificed by cervical dislocation on day 21. Blood was obtained by cardiac puncture. Spleen, tumor-draining lymph nodes and tumor were dissected. Tumors were cut into half for histologic and flow cytometric analysis. Animal experimentation was approved by the Regierungspräsidium Tübingen (permission number #1607).

### Sample preparation

Murine spleen and lymph nodes were dissociated using 40 µm nylon mesh. Tumors were dissociated using Mouse Tumor Dissociation kit (Miltenyi) and immune cells were isolated with EasySep™ Mouse TIL (CD45) Positive Selection kit (Stemcell). 1 × 10^6^ cells or 30 µl whole blood were used for flow cytometric analysis. Blood was additionally analyzed using VetScan HM5 (scil animal care company GmbH).

Human tumor and blood samples were obtained from the Department of Otorhinolaryngology, Ulm University Medical Center and processed using Human Tumor Dissociation kit, followed by isolation of immune cells using CD45 (TIL) MicroBreads (both Miltenyi). Peripheral blood mononuclear cells (PBMCs) were isolated from venous blood using Leukosep density centrifugation (Greiner Bio-One).

### Flow cytometry

After Fc-receptor blocking (Miltenyi) and staining with fixable viability dye (FVS) 700/780 (BD Biosciences), surface staining was performed for 30 min on ice (list of antibodies in supplementary Table 1). Murine blood samples were additionally treated with lysing solution (BD Biosciences). Samples were analyzed at Cytek Aurora or Beckman Coulter Gallios. Data was analyzed using FlowJo v10. Exemplary gating schemes are depicted in supplementary Figs. 1 and 2.

**Fig. 1:**
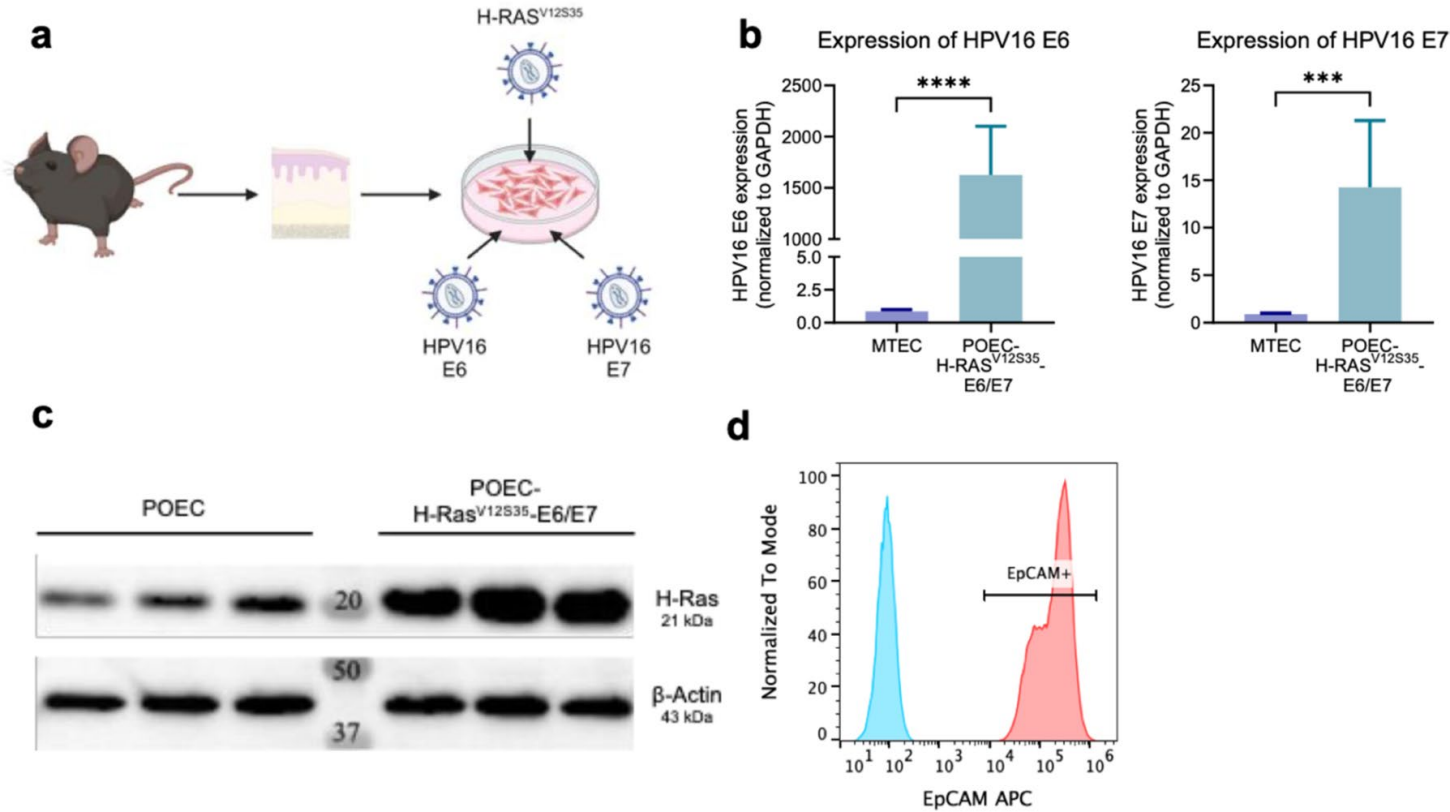
Generation of a murine cell line to mimic HPV-positive HNSCC. Murine primary oral epithelial cells (POECs) were isolated from oral mucosal tissue of wildtype C56BL/6 mice and retrovirally transduced for the overexpression of H-Ras^V12S35^ and E6/E7 of the human papilloma virus type 16 (HPV16) genome. **a** Schematic overview of the procedure (created by BioRender.com). **b** RNA expression levels of HPV16-E6 (left) and E7 (right) in original and modified POECs analyzed by qPCR and normalized to the house keeping gene GAPDH. **c** Protein levels of H-Ras in three samples of original and modified POECs analyzed by immunoblotting. Note that wildtype H-Ras was detected due to a lack of a mutation-specific antibody. β-actin served as loading control. **d** Flow cytometric analysis of EpCAM expression on POEC- H-RAS.^V12S35^-E6/E7. Blue histogram shows unstained control. Statistical significance of differences was determined using unpaired Student’s t tests and is depicted as *p* < 0.001 (***), *p* < 0.0001 (****)

**Fig. 2:**
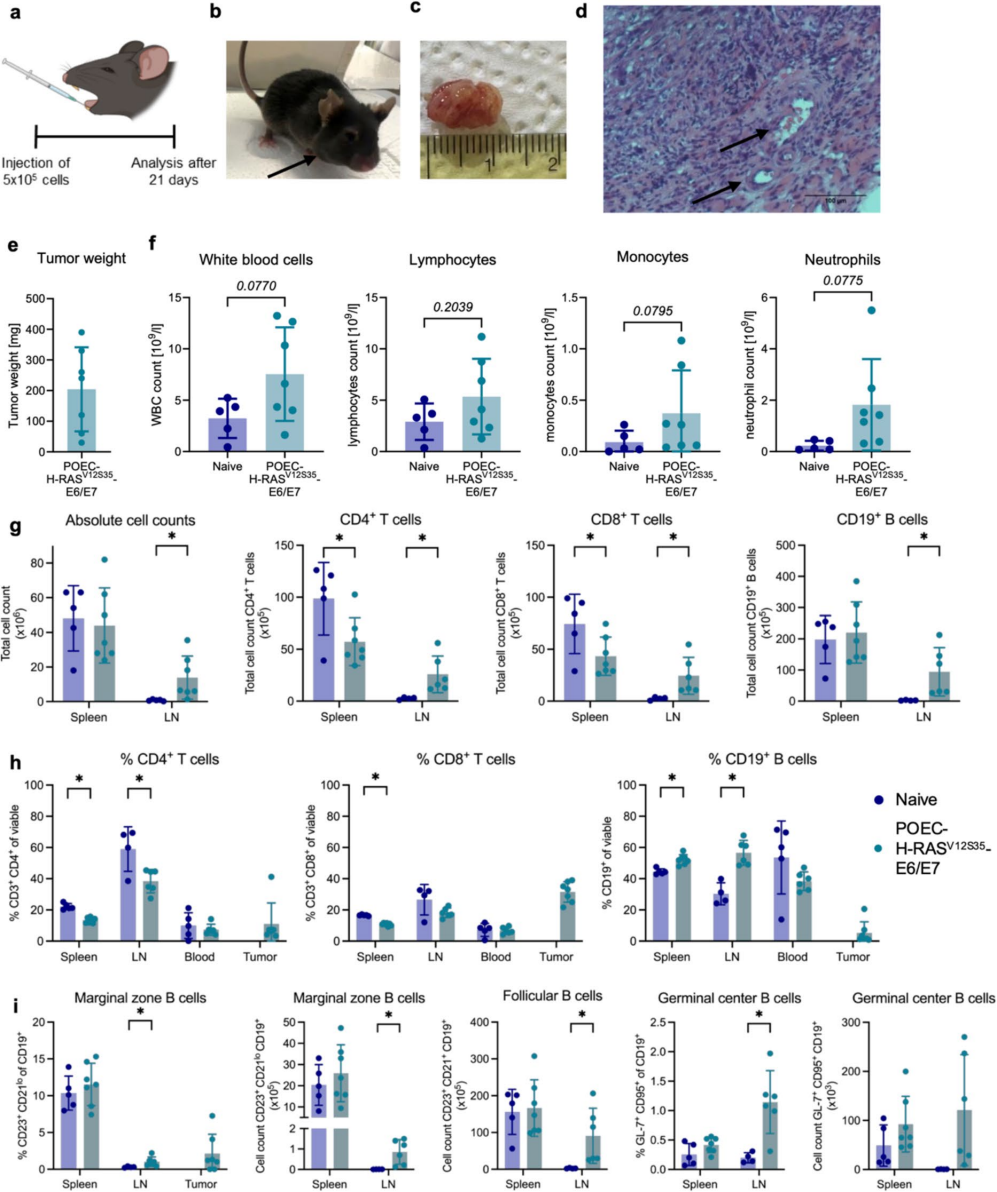
Characterization of T and B cell responses in POEC- H-RAS^V12S35^-E6/E7 tumor-bearing mice. 5 × 10^5^ POEC- H-RAS^V12S35^-E6/E7 cells were implanted into the floor of the mouth of C57BL/6 mice. Tumor growth and the immune response was analyzed after 21 days. **a** Experimental setup (created by BioRender.com). **b** Representative photograph of a tumor-bearing mouse on day 21 before analysis. **c** Representative pictures of dissected tumors and **d** micrographs of hematoxylin/eosin-stained tumor tissue. Arrows indicate blood vessels. **e** Tumor weight of dissected tumors. **f** Blood analysis using VetSan HM5 of naïve vs. tumor-bearing for the analysis of white blood cells (WBC), lymphocytes, monocytes and neutrophils. **g** Absolute cell counts of all cells and CD4^+^ and CD8^+^ T cells as well as CD19^+^ B cells in the spleen and lymph nodes in naïve compared to tumor-bearing mice determined by flow cytometry. **h** Relative proportions of T and B cells as well as **i** different B cells subsets in spleen, lymph node (LN), blood and tumor determined by flow cytometry. All cellular subsets were identified as specified on the y-axis of the graphs after gating on the lymphocyte population and exclusion of debris and dead cells. Data are shown as mean ± SD, and each point represents an individual mouse (n = 4–7). Statistical significance of differences between naïve and tumor-bearing mice was determined by unpaired Student’s t tests or Mann–Whitney U test for parametric or nonparametric data, respectively. Statistical significance is depicted as *p* < 0.05 (*) or given as full numbers of exact p-values

### Histology

Dissected tumors were immediately fixed in 4% formalin and subsequently embedded in paraffin. 5 µm sections were stained with hematoxylin and eosin and imaged at the Axio Observer (Zeiss).

### Statistical analysis

Statistical analysis and graphical representation of data were performed using GraphPad Prism version 10. Data was tested for normal distribution and subsequently analyzed using unpaired Student’s t tests or Mann–Whitney-U-test for parametric and non-parametric data, respectively. Statistical significance is depicted as *p* < 0.05 (*), *p* < 0.01 (**), *p* < 0.001 (***), and *p* < 0.0001 (****).

## Results

### Establishment of POEC-H-RAS^V12S35^-E6/E7 mimicking HPV-positive HNSCC

Preclinical mouse models provide an important platform for studying the complex interplay between immune system and tumors but only a few models are currently available. Following the example of Hoover et al. [7], we aimed at mimicking HPV-positive HNSCC by overexpressing the HPV16-oncogenes *E6* and *E7* in primary oral epithelial cells (POEC) combined with a constitutively active form of H-Ras (H-Ras^V12/S35^) since this pathway is often enhanced in HNSCC (Fig. 1a) [4, 10]. Successful overexpression of *E6/E7* and H-Ras in POEC-H-RAS^V12S35^-E6/E7 was confirmed on RNA and protein level, respectively (Fig. 1b + c). The epithelial origin of transduced cells was confirmed by strong EpCAM expression (Fig. 1d).

### Robust induction of the adaptive immune response in a mouse model of HPV-positive HNSCC

Since the adaptive immune response plays an important role in HPV-positive HNSCC, we analyzed T and B cell responses after 3 weeks of orthotopic tumor growth of POEC-H-RAS^V12S35^-E6/E7 (Fig. 2a + b). Tumors were induced in all mice even though tumor size (Fig. 2c + e) differed between the individual mice. Histologic analysis showed that also vascularization occurred within this time (Fig. 2d). Blood analysis suggested the induction of an active immune response seen as a tendency of increased cell counts for various subsets of immune cells (Fig. 2f). More strikingly, absolute cell counts of draining lymph nodes increased significantly compared to naïve mice which resulted from an increase in T and B cells (Fig. 2g). Interestingly, the total amounts of CD4^+^ and CD8^+^ T cells decreased in the spleen while increasing in lymph nodes, suggesting a recruitment to the site of tumor development (Fig. 2g + h) and T and B cells infiltrated the tumor, especially CD8^+^ T cells. Moreover, a strong B cell response was induced in the lymph nodes which were mainly follicular B cells. The amount of germinal center and marginal zone B cells also increased, suggesting that B cells play a critical role in the anti-tumor immune response (Fig. 2i).

### Immunoregulatory pathways are enhanced in mice bearing HPV-positive HNSCC tumors

**Fig. 3:**
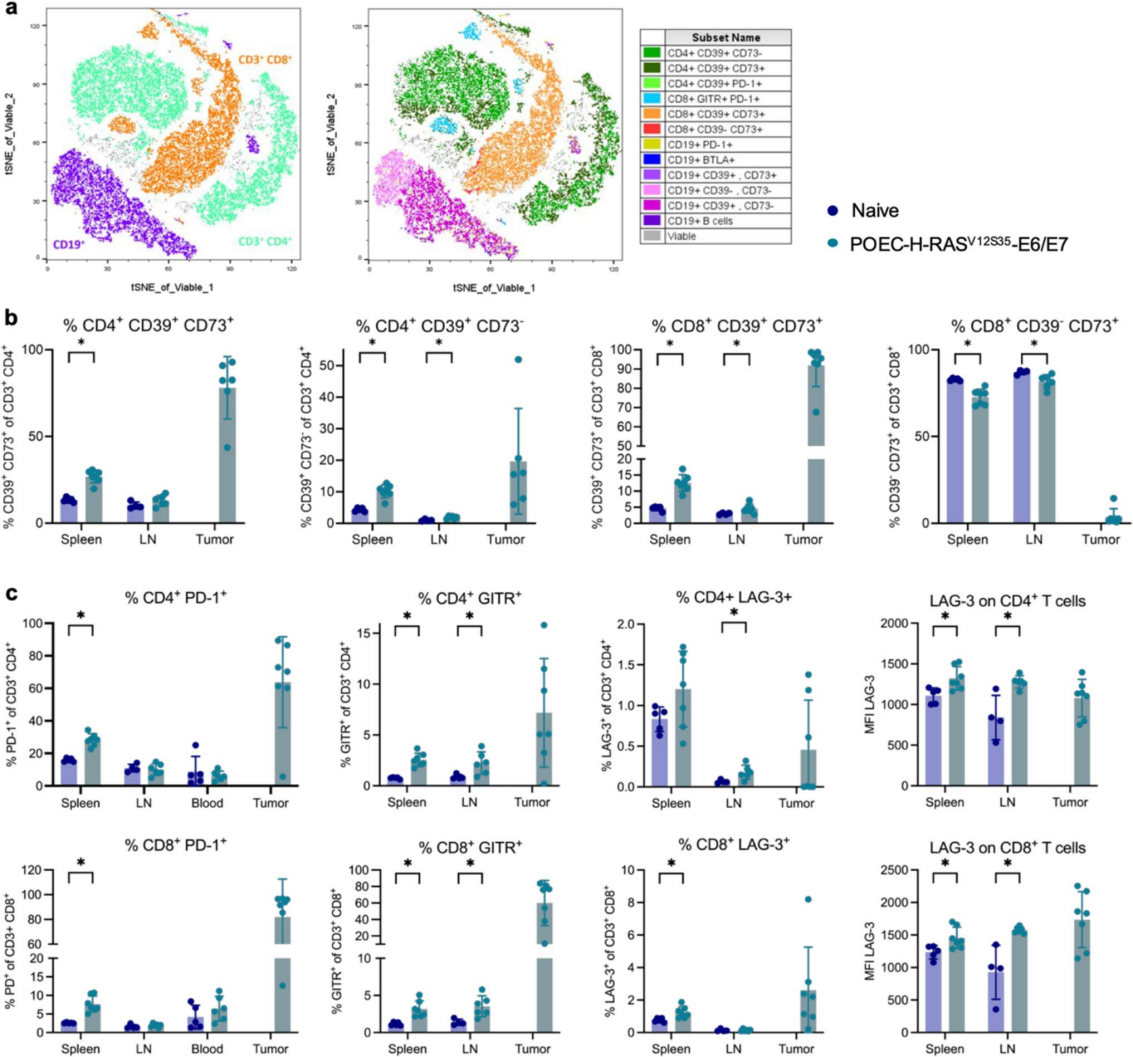
Immunoregulatory pathways are enhanced in POEC-H-RAS^V12S35^-E6/E7 tumor-bearing mice. 5 × 10^5^ POEC- H-RAS^V12S35^-E6/E7 cells were implanted into the floor of the mouth of C57BL/6 mice, and the immune response was analyzed after 21 days. **a** Representative tSNE blots of viable immune cells in POEC- H-RAS^V12S35^-E6/E7 tumors analyzed by spectral flow cytometry. Different immune cell populations were identified by traditional gating schemes and overlayed in different colors to visualize population distributions (legend on the right). **b** CD39 and/or CD73-expressing T cell subsets in spleen, lymph node (LN) and tumor. **c** CD4^+^ (upper panel) and CD8^+^ (lower panel) T cell subsets expressing various immunoregulatory receptors in the different organs. Data is shown as relative proportion of positive cells for all subsets and additionally as mean fluorescence intensity (MFI) for LAG-3 (right side). All cellular subsets were identified as specified on the y-axis of the graphs after gating on the lymphocyte population and exclusion of debris and dead cells. Data are shown as mean ± SD, and each point represents an individual mouse (n = 4–7). Statistical significance of differences between naïve and tumor-bearing mice was determined by unpaired student’s t tests or Mann–Whitney U test for parametric or nonparametric data, respectively, and depicted as *p* < 0.05 (*)

We further characterized the T cell response which is of great interest for potential immunotherapies. We observed strong induction of CD39 and/or CD73 on CD4^+^ and CD8^+^ T cells, a pathway for production of immunosuppressive adenosine (Fig. 3a + b). While only about 12% and 5% of CD4^+^ T cells express CD39 and CD73 or CD39 alone in the spleen of naïve mice, respectively, these proportions double in spleens of tumor-bearing mice. Strikingly, almost all CD4^+^ T cells inside the tumor express at least CD39 alone but mostly in combination with CD73. Similarly, CD8^+^ T cells, which mainly express CD73 alone in naïve mice, acquire CD39/CD73 double expression inside the tumor and to a smaller extent in the spleen and lymph nodes in tumor-bearing mice. Moreover, expression of the immune checkpoint receptor PD-1 was very abundant on both T cell subsets inside the tumor and significantly increased in the spleen of tumor-bearing mice (Fig. 3c), suggesting an exhausted phenotype of these cells. In line, expression of LAG-3, another immune checkpoint receptor, was enhanced in tumor-bearing mice even though to a lower extent than PD-1. Expression of the co-stimulatory receptor GITR was increased in all analyzed tissues, especially on CD8^+^ T cells which suggests T cell activation (Fig. 3c).

### Similar immunoregulatory pathways are found in human HPV-positive HNSCC

**Fig. 4:**
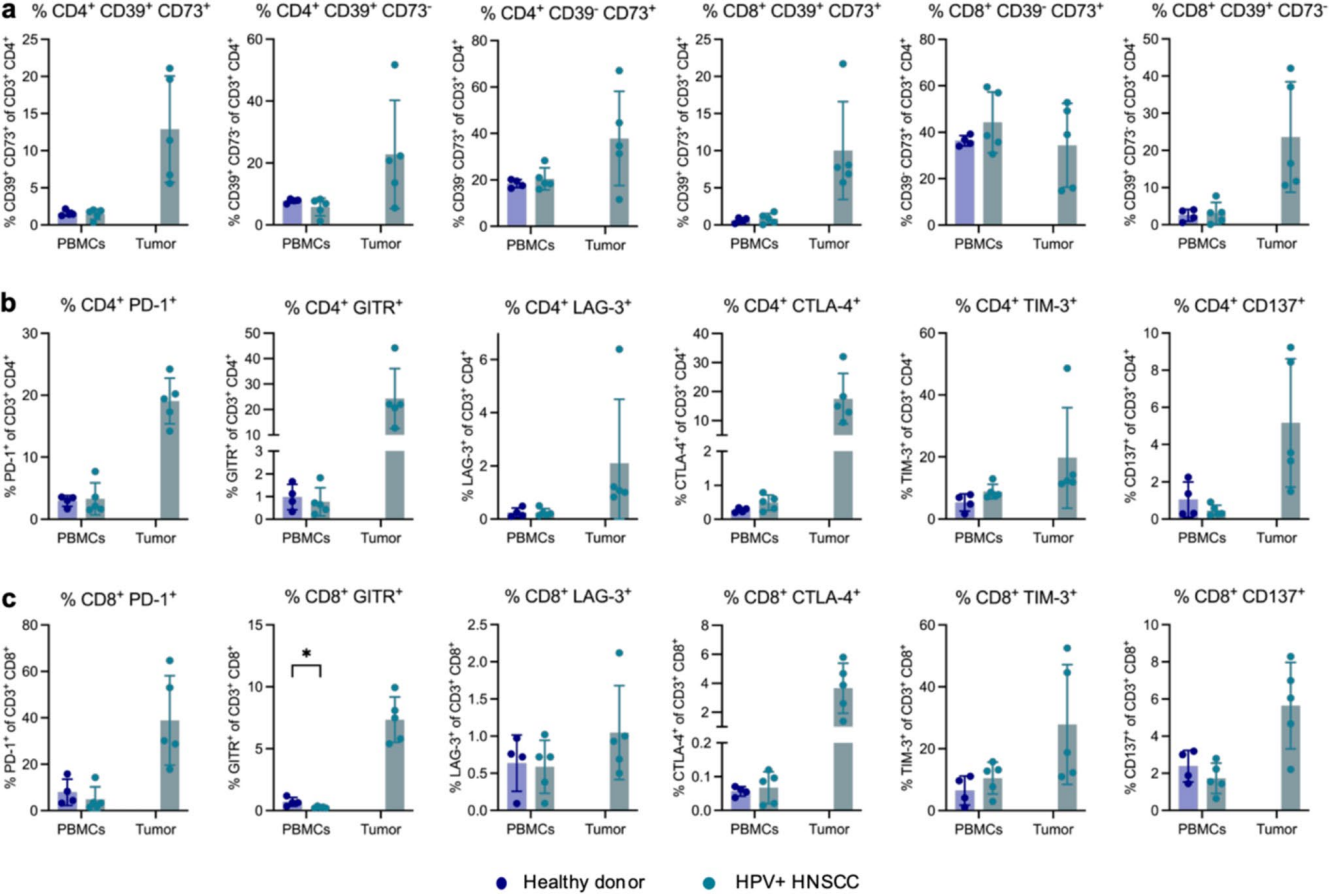
Immunoregulatory pathways in human HPV + HNSCC. Peripheral blood mononuclear cells (PBMCs) were obtained from healthy donors, and patients with human papilloma virus-positive (HPV +) HNSCC and tumor-infiltrating immune cells were isolated from tumors of the same HNSCC patients. Samples were analyzed by spectral flow cytometry and **a** several T cell subsets expressing CD39 and/or CD73 were identified. Relative proportion of **b** CD4^+^ and **c** CD8.^+^ T cells expressing various immunoregulatory receptors were identified among PBMCs and in the tumor. All cellular subsets were identified as specified on the y-axis of the graphs after gating on the lymphocyte population and exclusion of debris and dead cells. Data are shown as mean ± SD, and each point represents an individual donor (n = 4–5). Statistical significance of differences between heathy donors and HPV + HNSCC patients was determined by unpaired student’s t tests or Mann–Whitney U test for parametric or nonparametric data, respectively, and depicted as *p* < 0.05 (*)

Since we aimed at generating and characterizing a mouse model for preclinical studies of human HPV-positive HNSCC, we analyzed the same immunosuppressive pathways in tumor-infiltrating lymphocytes and PBMCs from patients with HPV-positive HNSCC compared to healthy controls (Supplementary Table 2). Similar to mice, CD39/CD73 co-expression on CD4^+^ and CD8^+^ T cells was higher in the tumor compared to PBMCs, even though to a lesser extent (Fig. 4a). Moreover, both cell types also partially expressed CD39 or CD73 alone which increased inside the tumor in most subsets. In line with the observations in mice, the proportions of PD-1^+^, GITR^+^ and LAG-3^+^ cells increased among CD4^+^ and CD8^+^ T cells in the tumor compared to the blood (Fig. 4b + c). Additionally, we found increases in CTLA-4, TIM-3 and CD137 expression among T cells in the tumor compared to PBMCs, suggesting a complex immunoregulatory network inside the tumor with several possibilities for therapeutical interference.

## Discussion

Preclinical models for HNSCC are required to better understand immunologic and oncologic processes to identify urgently needed novel therapeutic targets. Here, we could show that orthotopic tumor growth of our generated cell line overexpressing *E6/E7* of HPV16 in combination with the constitutively active H-Ras^V12S35^-mutant induces strong adaptive immune responses within three weeks of tumor growth. This is in line with human disease since especially HPV-positive HNSCC is characterized by strong immune infiltration [11] which we could confirm in our cohort of cancer patients. Changes in immune cell counts and expression of immunoregulatory markers was not limited to the tumor itself but could be observed systemically in secondary lymphoid organs and the blood. In line with the literature [12, 13], we observed increased levels of PD-1^+^, GITR^+^ and LAG-3^+^ T cells in the tumor and in the periphery of tumor-bearing mice. Similar patterns were detected in human tumor samples along with expression of additional immunoregulatory molecules such as CTLA-4, TIM-3 and CD137 (4-1BB) that could be detected on more T cells in the tumor than in the periphery. GITR and 4-1BB are co-stimulatory molecules which are upregulated upon T cell activation. PD-1, LAG-3, TIM-3 and CTLA-4 exhibit inhibitory functions characteristic of regulatory and/or exhausted T cells, partially linked to worse prognosis in HNSCC patients [13, 14]. Upregulation of immunosuppressive pathways is a physiologic negative-feedback mechanism of any immune response, indicating T cell activation and a robust initial anti-tumor response. Therefore, high frequency of infiltrating regulatory T cells can serve as marker for a strong immune response and is associated with better prognosis, e.g., shown for PD-1 expression in HPV-positive HNSCC [15–17]. Nevertheless, the resulting immunosuppressive environment will impact the sufficient anti-tumor response at a later stage and thus serves as a potential therapeutic target.

Similarly, we observed strong expression of CD39 and CD73 on almost all tumor-infiltrating T cells. CD39 and CD73 are ectoenzymes that convert extracellular ATP into AMP and adenosine. Adenosine has immunosuppressive functions shown to be employed by regulatory immune cell subsets such as regulatory T and B cells [11, 18, 19]. 

Besides changes in T cell populations, we also observed an altered B cell response in tumor-bearing mice. B cells are implicated to play a beneficial role in the anti-tumor response, for example by forming germinal centers inside the tumor, which is associated with improved survival [20]. Probably due to the comparably short time frame of tumor growth in our setting, infiltration of B cells into the tumor was not as strong as seen for T cells but an increase in germinal center and follicular B cells was observed in spleen and lymph node of tumor-bearing mice. Modification of experimental design could be interesting in future studies to investigate the B cell response. Longer time or changing injection site would further allow analysis of invasive growth into surrounding tissue or enable comparative studies to determine differences between tumor locations. Moreover, establishment of cell lines carrying different mutations could be useful to compare their impact on immune responses.

Taken together, our data show that the induced adaptive immune response in this HPV-positive HNSCC mouse model shares characteristics with human disease and can thus be used as a platform to study anti-tumor responses which will help to identify novel therapeutic targets.

## Supplementary Information

Below is the link to the electronic supplementary material.

Supplementary file1 (PDF 401 KB)

## Data Availability

No datasets were generated or analyzed during the current study.

## Abbreviations

FCS: Fetal calf serum
HD: Healthy donor
HNSCC: Head and neck squamous cell carcinoma
HPV: Human papilloma virus
POEC: Primary oral epithelial cells
PBMCs: Peripheral blood mononuclear cells
TILs: Tumor-infiltrating lymphocytes

## References

[1] Lechner M, Liu J, Masterson L, Fenton TR (2022) HPV-associated oropharyngeal cancer: epidemiology, molecular biology and clinical management. Nat Rev Clin Oncol 19:306–327. 10.1038/s41571-022-00603-7 35105976 10.1038/s41571-022-00603-7 PMC8805140

[2] Gillison ML, D’Souza G, Westra W et al (2008) Distinct risk factor profiles for human papillomavirus type 16–positive and human papillomavirus type 16–negative head and neck cancers. JNCI J Nat Cancer Inst 100:407–420. 10.1093/jnci/djn025 10.1093/jnci/djn025 18334711

[3] Lawrence MS, Sougnez C, Lichtenstein L et al (2015) Comprehensive genomic characterization of head and neck squamous cell carcinomas. Nature 517:576–582. 10.1038/nature14129 25631445 10.1038/nature14129 PMC4311405

[4] Johnson DE, Burtness B, Leemans CR et al (2020) Head and neck squamous cell carcinoma. Nat Rev Dis Primers 6:92. 10.1038/s41572-020-00224-3 33243986 10.1038/s41572-020-00224-3 PMC7944998

[5] Ferris RL (2015) Immunology and immunotherapy of head and neck cancer. J Clin Oncol 33(3293):3304. 10.1200/JCO.2015.61.1509 10.1200/JCO.2015.61.1509 PMC4586169 26351330

[6] Burtness B, Harrington KJ, Greil R et al (2019) Pembrolizumab alone or with chemotherapy versus cetuximab with chemotherapy for recurrent or metastatic squamous cell carcinoma of the head and neck (KEYNOTE-048): a randomised, open-label, phase 3 study. The Lancet 394:1915–1928. 10.1016/S0140-6736(19)32591-7 10.1016/S0140-6736(19)32591-7 31679945

[7] Hoover AC, Spanos WC, Harris GF et al (2007) The role of human papillomavirus 16 E6 in anchorage-independent and invasive growth of mouse tonsil epithelium. Arch Otolaryngol Head Neck Surg 133:495–502. 10.1001/archotol.133.5.495 17515506 10.1001/archotol.133.5.495 PMC2917346

[8] Shivarudrappa AH, John J, Vashisht M, et al (2024) Differential tumor immune microenvironment coupled with tumor progression or tumor eradication in HPV-antigen expressing squamous cell carcinoma (SCC) models. Front Immunol 15:10.3389/fimmu.2024.1405318 PMC11269233 39055715

[9] Leichtle F, Betzler AC, Eizenberger C, et al (2023) Influence of Bruton’s Tyrosine Kinase (BTK) on epithelial–mesenchymal transition (EMT) processes and cancer stem cell (CSC) enrichment in head and neck squamous cell carcinoma (HNSCC). Int J Mol Sci 24:. 10.3390/ijms241713133 10.3390/ijms241713133 PMC10487612 37685940

[10] Kiaris H, Spandidos DA, Jones AS et al (1995) Mutations, expression and genomic instability of the H-ras proto-oncogene in squamous cell carcinomas of the head and neck. Br J Cancer 72:123–128. 10.1038/bjc.1995.287 7599040 10.1038/bjc.1995.287 PMC2034114

[11] Ruffin AT, Li H, Vujanovic L et al (2023) Improving head and neck cancer therapies by immunomodulation of the tumour microenvironment. Nat Rev Cancer 23:173–188. 10.1038/s41568-022-00531-9 36456755 10.1038/s41568-022-00531-9 PMC9992112

[12] Lechner A, Schlößer H, Rothschild SI, et al (2017) Characterization of tumor-associated T-lymphocyte subsets and immune checkpoint molecules in head and neck squamous cell carcinoma. Oncotarget 8:44418–44433. 10.18632/oncotarget.17901 10.18632/oncotarget.17901 PMC5546490 28574843

[13] Damasio MPS, Nascimento CS, Andrade LM, et al (2022) The role of T-cells in head and neck squamous cell carcinoma: From immunity to immunotherapy. Front Oncol 12 10.3389/fonc.2022.1021609 PMC9632296 36338731

[14] Liu Z, McMichael EL, Shayan G et al (2018) Novel effector phenotype of tim-3+ regulatory T cells leads to enhanced suppressive function in head and neck cancer patients. Clin Cancer Res 24:4529–4538. 10.1158/1078-0432.CCR-17-1350 29712685 10.1158/1078-0432.CCR-17-1350 PMC6139056

[15] Seminerio I, Descamps G, Dupont S, et al (2019) Infiltration of FoxP3+ regulatory T cells is a strong and independent prognostic factor in head and neck squamous cell carcinoma. Cancers (Basel) 11. 10.3390/cancers11020227 10.3390/cancers11020227 PMC6406934 30781400

[16] Badoual C, Hans S, Merillon N et al (2013) PD-1–expressing tumor-infiltrating T cells are a favorable prognostic biomarker in HPV-associated head and neck cancer. Cancer Res 73:128–138. 10.1158/0008-5472.CAN-12-2606 23135914 10.1158/0008-5472.CAN-12-2606

[17] Julian R, Savani M, Bauman JE (2021) Immunotherapy approaches in HPV-associated head and neck cancer. Cancers (Basel) 13:. 10.3390/cancers13235889 10.3390/cancers13235889 PMC8656769 34884999

[18] Saze Z, Schuler PJ, Hong C-S et al (2013) Adenosine production by human B cells and B cell-mediated suppression of activated T cells. Blood 122:9–18. 10.1182/blood-2013-02-482406 23678003 10.1182/blood-2013-02-482406 PMC3701906

[19] Jie H-B, Gildener-Leapman N, Li J et al (2013) Intratumoral regulatory T cells upregulate immunosuppressive molecules in head and neck cancer patients. Br J Cancer 109:2629–2635. 10.1038/bjc.2013.645 24169351 10.1038/bjc.2013.645 PMC3833228

[20] Ruffin AT, Cillo AR, Tabib T et al (2021) B cell signatures and tertiary lymphoid structures contribute to outcome in head and neck squamous cell carcinoma. Nat Commun 12:3349. 10.1038/s41467-021-23355-x 34099645 10.1038/s41467-021-23355-x PMC8184766

