# Supplementary material for: Characterization of the adaptive immune response in a mouse model for HPV-positive head and neck squamous cell carcinoma with implications to human disease
Source: Cancer Immunol Immunother. 2025 Jan 3;74(2):66. doi: 10.1007/s00262-024-03907-y (PMC11698698; doi:10.1007/s00262-024-03907-y)
Supplement: Supplementary file 1 — Supplementary file1 (PDF 401 KB) [file 262_2024_3907_MOESM1_ESM.pdf]

**Human antigen**	**Fluorochrome**	**Order number**	**Company**
CD3	cFluor V420	R7-20053	Cytek
CD8	BV650	344730	Biolegend
CD4	SparkViolet538	344674	Biolegend
CD19	cFluor BYG710	R7-20009	Cytek
CD39	PE-Fire810	328245	Biolegend
CD73	BUV805	748584	BD Biosciences
CD25	AlexaFluor700	302622	Biolegend
CD69	APC	310910	Biolegend
CD44	BV785	338834	Biolegend
CD30	PE	333906	Biolegend
PD-1	PerCP-Cy5.5	329914	Biolegend
PD-L1 (CD274)	PE	329706	Biolegend
CTLA-4 (CD152)	PE-Cy5	555854	BD Biosciences
CD86	AF700	561124	BD Biosciences
HVEM (CD270)	BUV737	748637	BD Biosciences
GITR (CD357)	BV605	371214	Biolegend
LAG3 (CD223)	Alexa Fluor488	369326	Biolegend
TIM3 (CD366)	PE-CF594	565560	BD Biosciences
OX40 (CD134)	BV480	746511	BD Biosciences
OX40L (CD252)	APC	108812	Biolegend
HLA-DR	BUV496	749866	BD Biosciences
CD137	BV711	309832	Biolegend
**Mouse antigen**			
CD3	V500	560771	BD Biosciences
CD8	FITC	11-0081-82	Invitrogen
CD4	PercP	553052	BD Biosciences
CD19	APC-Fire750	115558	Biolegend
CD39	PE-Cy7	25-0391-82	Invitrogen
CD73	BV605	127215	Biolegend
PD-1	PE	12-9985-82	Invitrogen
CD152 (CTLA-4)	PerCP-Cy5.5	106316	Biolegend
CD272 (BTLA)	BV650	740640	BD Biosciences
CD223 (LAG-3)	BUV395	745693	BD Biosciences
CD27	BV785	124241	Biolegend
CD357 (GITR)	BV421	563391	BD Biosciences
CD23	PE-Dazzle594	751169	BD Biosciences
CD95	BUV615	101634	Biolegend
GL7	AlexaFluor647	561529	BD Biosciences
CD21	BUV737	612810	BD Biosciences
EpCAM	APC	118213	Biolegend

Supplementary Table 1: List of antibodies for flow cytometry.

	**HNSCC patients (n=5)**	**Healthy donors (n=4)**
**Age** (mean ± SD)	62,20 ± 4,764	55,75 ± 4,573
**Sex**		
male	5	4
female	0	0
**Location**		
Oropharynx	5	
**HPV status**		
p16+	5	
HPV16-DNA+	5	
not tested	0	4
**T status**		
1	0	
2	4	
3	1	
4	0	
**Nodal status**		
N0	1	
N+	4	
**UICC stage**		
I	0	
II	0	
III	5	
IV	0	

Supplementary Table 2: Patient and healthy donor characteristics.

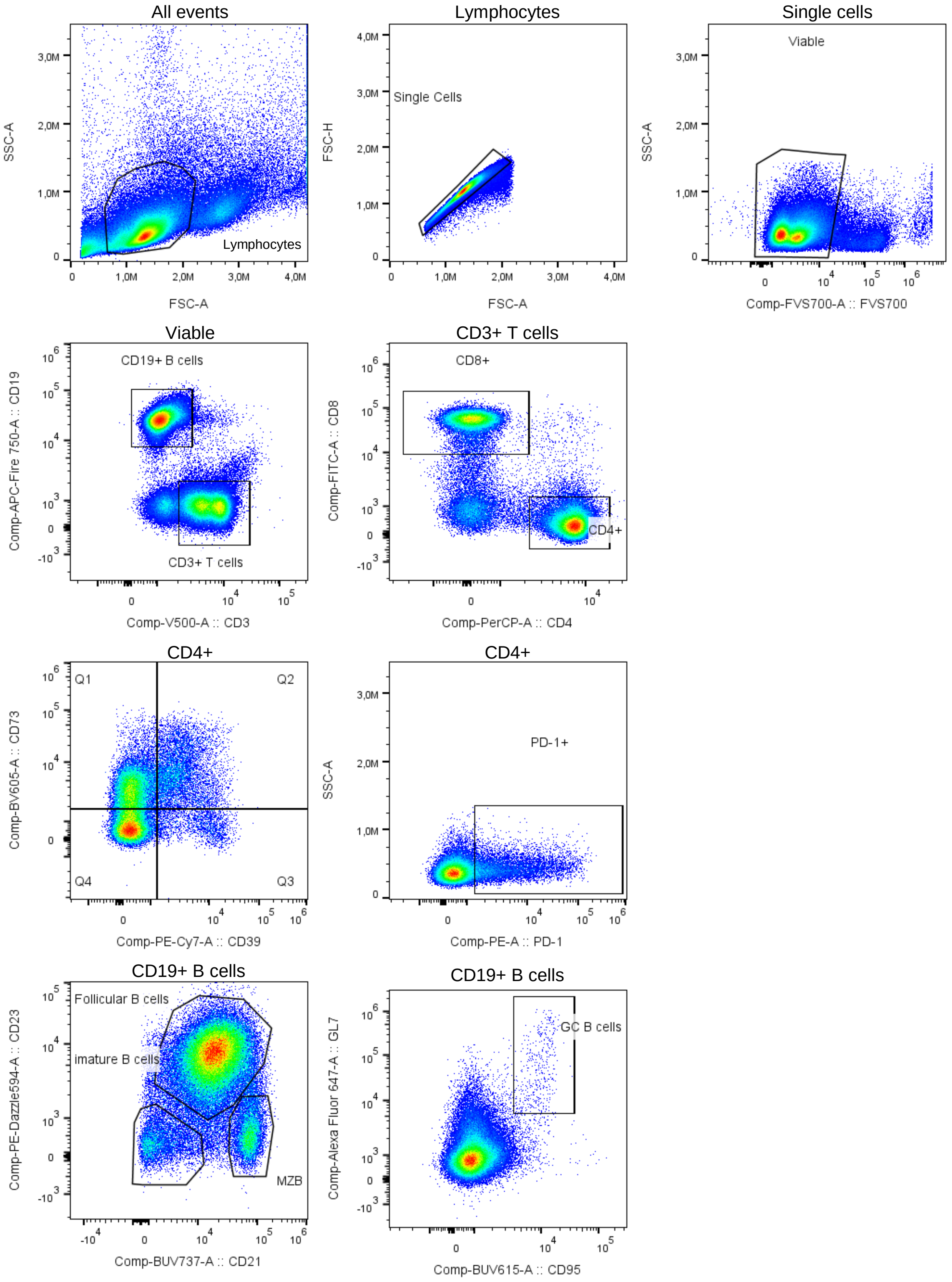

Supplementary Figure 1: Gating scheme for murine lymphocytes. Single cell suspensions of spleen, lymph nodes, blood and tumor were analyzed by spectral flow cytometry. A representative gating scheme is shown from the spleen. All samples were first gated on the lymphocyte population according to the size and granularity of the cells. Next, doublets and dead cells (positive for fixable viability dye (FVS) 700) were excluded. For blood samples, erythrocyte exclusion was included by assessing side scatter characteristics on the violet and blue laser (not shown). B and T cells were identified by their expression of CD19 and CD3, respectively. T cells were further divided into CD4 or CD8 positive and CD39/CD73 expression as well as several immunoregulatory receptors such as PD-1 were analyzed (exemplarily shown for CD4+). B cell subsets of follicular, immature or marginal zone B cells (MZB) were identified by their expression of CD21/CD23 and germinal center (GC) B cells as CD95^{+} GL-7^{+}.

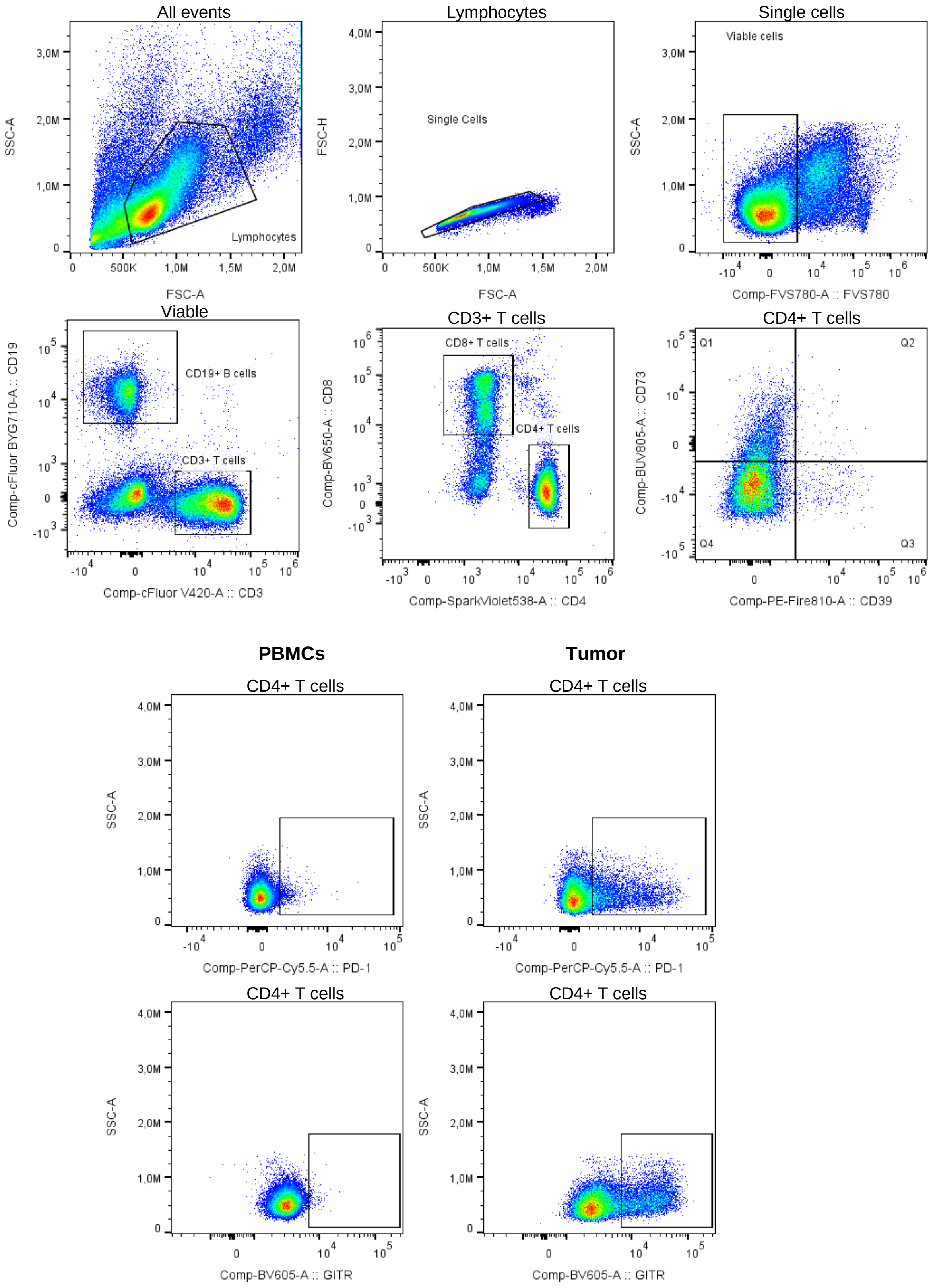

Supplementary Figure 2: Gating scheme for human lymphocytes. Peripheral blood mononuclear cells (PBMCs) and tumor infiltrating immune cells were analyzed by spectral flow cytometry. Upper two panels: a representative gating scheme is shown for PBMCs. All samples were first gated on the lymphocyte population according to the size and granularity of the cells. Next, doublets and dead cells (positive for fixable viability dye (FVS) 780) were excluded. B and T cells were identified by their expression of CD19 and CD3, respectively. T cells were further divided into CD4 or CD8 positive and CD39/CD73 expression were analyzed. Lower panel: several immunoregulatory receptors such as GITR and PD-1 were subsequently analyzed, here exemplarily shown for CD4+ T cells among PBMCs and in the tumor.
